# Beyond the Textbook: AI’s Role in Shaping the Future of Psychiatric Education

**DOI:** 10.1192/bjo.2026.11324

**Published:** 2026-06-30

**Authors:** Jasleen Deol, Tejbir Deol

**Affiliations:** 1 Aston University, Birmingham, United Kingdom; 2 Black Country Healthcare NHS Foundation Trust, Dudley, United Kingdom; 3 GKT School of Medical Education, Kings College London, London, United Kingdom

## Abstract

**Aims:**

Different teaching modalities are used within medical curricula to educate students about the principles and intricacies of psychiatry. Given the advancement in digital technology, medical education is evolving to include new digital based interventions and learning approaches, including the use of artificial intelligence (AI) based interventions. This review aims to systematically identify how artificial intelligence is used within medical curricula when teaching students about psychiatry, and is unique as reviewers are at different stages of training.

**Methods:**

An advanced literature search was undertaken using OVID (MEDLINE), and Web of Science. Four key search terms were used within these databases – ‘psychiatry*’ AND ‘medical education’ AND ‘artificial intelligence’ AND ‘students’. Studies commenting or focusing on artificial intelligence-based interventions within healthcare education were included, and no restriction on the country, language or methodology. PRISMA checklist utilised. Results were thematically analysed.

**Results:**

332 studies were retrieved (19 were excluded due to duplication). 313 studies were screened, and 16 full texts were screened and 4 studies were deemed suitable for inclusion.

Key areas highlighted within these studies suggest AI can be in a versatile manner to help shape educational interventions. One study highlighted numerous roles that ChatGPT can undertake within an educational setting, including providing prompts for debates within students, facilitating self-directed learning, providing information to students, and can be used to create further learning materials, including vignettes for hypothetical cases – after a relevant prompt. However, the study highlighted key limitations to consider when using this novel approach as follows: the likelihood of inaccuracies leading to misinformation, differences between languages/translation, and lackof replicability and reproducibility of responses and results.

Another study showcased the creation of a web-based AI supported educational tool developed for psychiatric education, and students reported significant user satisfaction and mentioned that the platform was effective and supported them through their placements.

**Conclusion:**

This review highlights the limited availability of literature surrounding the use of artificial intelligence to teach medical students about psychiatry. Additionally, the limited available literature highlighted that AI can be used in a versatile manner to create learning prompts, aides, and to create realistic case studies and vignettes that can help improve learning. However several limitations have been highlighted about the use ofAI-related materials within medical education, and further research and innovation is required within this area.

